# H_2_O_2_-Based Method for Rapid Detection of Transgene-Free Rice Plants from Segregating CRISPR/Cas9 Genome-Edited Progenies

**DOI:** 10.3390/ijms20163885

**Published:** 2019-08-09

**Authors:** Tsung-Meng Wu, Jian-Zhi Huang, Hui-Min Oung, Yi-Ting Hsu, Yu-Chang Tsai, Chwan-Yang Hong

**Affiliations:** 1 Department of Agricultural Chemistry, College of Bioresources and Agriculture, National Taiwan University, Taipei 10617, Taiwan; 2 Department of Aquaculture, National Pingtung University of Science and Technology, Pingtung 91201, Taiwan; 3 Department of Agronomy, College of Agriculture and Natural Resources, National Chung-Hsing University, Taichung 10617, Taiwan; 4 Department of Agronomy, College of Bioresources and Agriculture, National Taiwan University, Taipei 10617, Taiwan

**Keywords:** DAB (3,3′-diaminobenzidine), H_2_O_2_, hygromycin, hygromycin phosphotransferase, rice, CRISPR, reactive oxygen species

## Abstract

Genome-editing techniques such as CRISPR/Cas9 have been widely used in crop functional genomics and improvement. To efficiently deliver the guide RNA and Cas9, most studies still rely on *Agrobacterium*-mediated transformation, which involves a selection marker gene. However, several limiting factors may impede the efficiency of screening transgene-free genome-edited plants, including the time needed to produce each life cycle, the response to selection reagents, and the labor costs of PCR-based genotyping. To overcome these disadvantages, we developed a simple and high-throughput method based on visual detection of antibiotics-derived H_2_O_2_ to verify transgene-free genome-edited plants. In transgenic rice containing *hygromycin phosphotransferase* (*HPT*), H_2_O_2_ content did not change in the presence of hygromycin B (HyB). In contrast, in transgenic-free rice plants with 10-h HyB treatment, levels of H_2_O_2_ and malondialdehyde, indicators of oxidative stress, were elevated. Detection of H_2_O_2_ by 3,3′-diaminobenzidine (DAB) staining suggested that H_2_O_2_ could be a marker to efficiently distinguish transgenic and non-transgenic plants. Analysis of 24 segregating progenies of an *HPT*-containing rice plant by RT-PCR and DAB staining verified that DAB staining is a feasible method for detecting transformants and non-transformants. Transgene-free genome-edited plants were faithfully validated by both PCR and the H_2_O_2_-based method. Moreover, HyB induced overproduction of H_2_O_2_ in leaves of *Arabidopsis*, maize, tobacco, and tomato, which suggests the potential application of the DAB method for detecting transgenic events containing *HPT* in a wide range of plant species. Thus, visual detection of DAB provides a simple, cheap, and reliable way to efficiently identify transgene-free genome-edited and *HPT*-containing transgenic rice.

## 1. Introduction

The hygromycin phosphotransferase gene (*HPT*) from *E. coli* is a positive selection marker for plant transformation [1]. *HPT* confers resistance to hygromycin B (HyB) in transgenic plants by adding a phosphate to position seven of the destomic acid ring in HyB [1,2]. More importantly, HyB does not affect regeneration and fertility of transgenic plants [3]. Therefore, *HPT* is broadly used in the transformation of monocot plants, which usually show high levels of natural resistance to another antibiotic, kanamycin [4,5]. Plants effectively selected by using *HPT* include rice [6], maize [1], wheat [7,8], brachypodium [9,10], and barley [11,12,13].

HyB is an aminoglycoside antibiotic isolated from *Streptomyces hygroscopicus* [14] that has broad-spectrum activity against both prokaryotic and eukaryotic cells by interfering with translocation of the 70S ribosome and causing misreading of the mRNA template [15,16]. HyB significantly inhibits plant growth and development. Different plant genotypes exhibit variable HyB sensitivity. Culture media containing 50 mg L^−1^ HyB could fully inhibit the growth of rice callus [17] and cotyledon and leaves of *Arabidopsis* seedlings [18]; as low as 2.5 mg L^−1^ HyB was able to restrict the growth of maize cells [19].

Rice is a staple food crop feeding more than half the world’s population [20]. To accelerate biotechnological applications, the rice transformation system was established more than two decades ago [6,21]. During rice transformation, *HPT* is used for screening putative transgenic events and screening massive transgenic progenies or homozygous transgenic lines, thereby replacing the costly and labor-intensive PCR-based or Southern blot analysis [22,23,24]. For efficient HyB screening, seeds are germinated on medium containing HyB [24,25]; however, the germination rate may be confusing in seeds with dormant or low germination vigor, which affects the determination of homozygosity. 

CRISPR/Cas technology has emerged as a powerful and promising method to precisely modify the plant genome and efficiently generate transgene-free crops [26,27]. The introduction of a CRISPR/Cas-mediated genome-editing cassette into the plant genome allows for integrating the transgene into one locus and performing the editing at another locus. Therefore, traits can be segregated by sexual reproduction, generating progenies free of the transgene [26,27,28]. The transgene-free crops thus contain biallelic/homozygous mutations and are free of selection markers. A number of useful methods have been developed for screening biallelic/homozygous mutations. However, quick screening of marker-free transgenic plants from a genome-edited (GE) plant population remains a challenge because PCR-based screening is labor-intensive; furthermore, plants cannot survive on selection medium without selection markers. Thus, a reliable, inexpensive, and non-lethal selection method is needed to efficiently distinguish GE plants with or without selection markers.

Leaf painting assay has been used to facilitate the selection of transgenic plants tolerant to antibiotics or herbicides. In cotton, leaves of transgenic cotton treated with 750 mg/L kanamycin exhibited chlorosis after five to seven days and then necrotic patches after 10 days [29]. Transgenic rice [30] and maize [31] expressing the *bar* gene were able to tolerate the herbicide phosphinothricin. In transgenic maize, more than 95% of transgenic events can be verified by leaf painting assay, with results agreeing with PCR results [31]. Leaf painting assay is simple and efficient, but it takes almost one week to observe the wilt symptoms. Therefore, developing a simple, efficient, and rapid leaf painting assay is needed for high throughput screening of transgenic progenies.

3,3-Diaminobenzidine (DAB) staining is one of the most commonly used methods for H_2_O_2_ detection. After being taken up by plants, DAB reacts with H_2_O_2_ to form a dark-brown reaction product in the presence of peroxidase [32]. Our recent research indicated that HyB significantly and rapidly enhanced the accumulation of H_2_O_2_ in rice leaves [33]. Taking advantage of the high production of H_2_O_2_ in plants induced by HyB, we aimed to develop a simple and quick, selection-independent, H_2_O_2_-based assay system for identifying transgenic rice. 

In the present study, transgenic and non-transgenic rice could be easily distinguished by the H_2_O_2_-based assay system. The visual selection method provides a quick and reliable way for screening transgene-free GE plants after genome editing in rice. Moreover, we found HyB-induced overproduction of H_2_O_2_ in a wide range of plant species, so the H_2_O_2_ DAB method may be applicable for efficiently distinguishing a wide range of transgenic and non-transgenic plants.

## 2. Results

### 2.1. Hygromycin (HyB) Significantly Increased the Production of H_2_O_2_ in Leaves of Rice Seedlings

**Figure 1:**
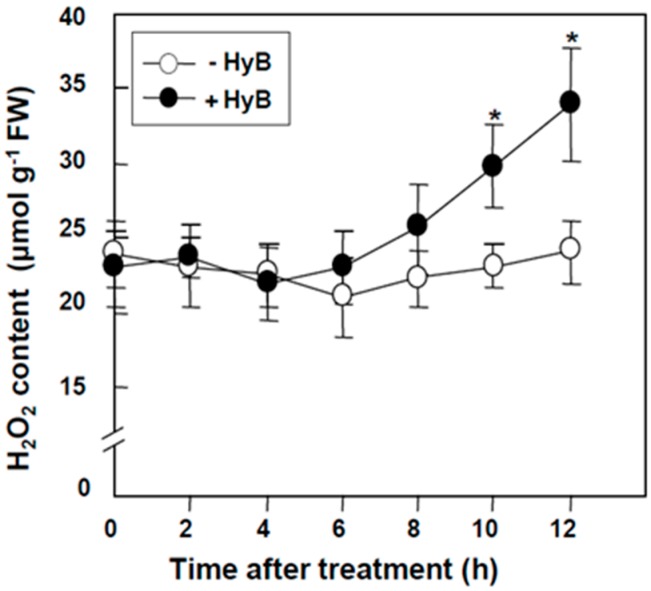
Temporal accumulation of hygromycin B (HyB)-induced hydrogen peroxide (H_2_O_2_) in rice leaves. Leaf segments of 2-week-old rice seedlings were incubated with or without 50 mg L^−1^ HyB for the indicated time. Data are mean ± SE (*n* = 4). * *P* < 0.05 compared with –HyB.

To examine the effect of HyB on the accumulation of H_2_O_2_, leaf segments of rice seedlings were treated with or without 50 mg mL^−1^ HyB for various times. H_2_O_2_ level increased with increasing treatment time. H_2_O_2_ level slightly increased after 6-h treatment and significantly increased after 10-h treatment (Figure 1). 

### 2.2. Accumulation of H_2_O_2_ in Transgenic Rice Overexpressing HPT

**Figure 2:**
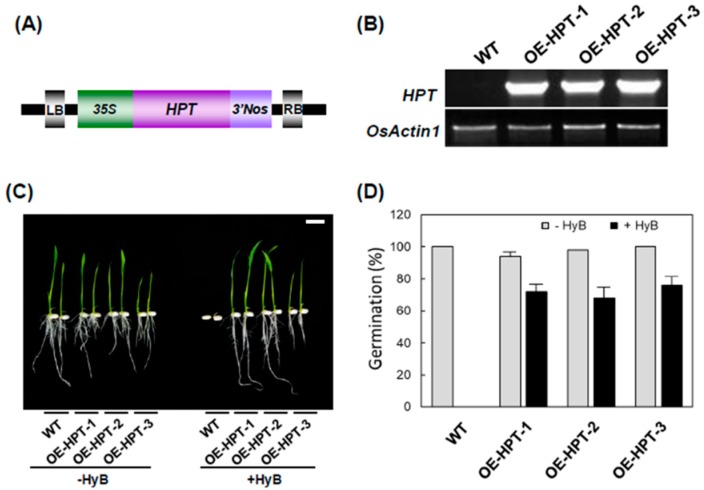
Generation of transgenic rice overexpressing hygromycin phosphotransferase. (**A**) Schematic representation of the *pPZP/HPH* binary vector used for rice transformation. 35S, Cauliflower mosaic virus 35S promoter; *HPT*, *hygromycin phosphotransferase* (*HPT*); *3’Nos,* 3’UTR of *Nopaline synthase.* (**B**) RT-PCR analysis of mRNA expression of *HPT* in wild-type (WT) and *HPT*-overexpressing rice plants. *OsActin1*, rice *actin1* gene. (**C**) Phenotypes of rice seedlings grown on hydroponic solution with (+HyB) or without (−HyB) 50 mg L^−1^ HyB for 7 days. (bar = 1 cm). (**D**) The number of germinated and non-germinated plants in WT and *HPT*-overexpressing rice plants grown on hydroponic solution with (+HyB) or without (−HyB) 50 mg L^−1^ HyB for 7 days (*n* = 50). Absolute percent germination is reported as the average of the mean of three independent experiments with the error bars representing SD.

**Figure 3:**
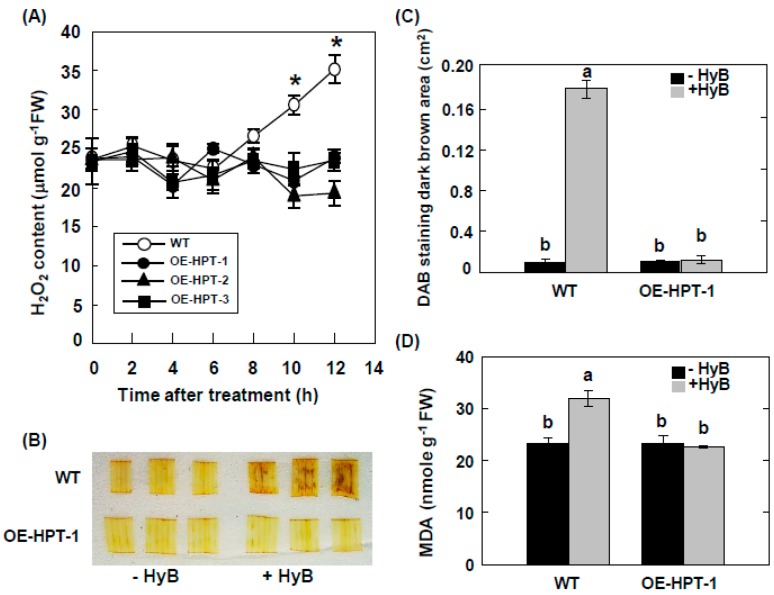
H_2_O_2_ content in WT and *HPT*-overexpressing seedlings under HyB treatment. (**A**) changes in H_2_O_2_ content in leaf segments of WT and *HPT*-overexpressing 2-week-old seedlings treated with 50 mg L^−1^ HyB for 12 h. Data are mean ± SE (*n* = 4). * *P* < 0.05 compared with WT. (**B**) Histochemical detection of H_2_O_2_ with DAB staining in leaf segments of WT and *HPT*-Overexpressing rice plants (OE-HPT-1) treated with or without HyB. (**C**) Quantification of DAB staining. The histogram tool in ImageJ was used to record the grayscale values of all pixels within the brown areas of eight-bit images. (**D**) Malondialdehyde (MDA) content in WT and *HPT*-overexpressing rice plants (OE-HPT-1) treated with or without 50 mg L^−1^ HyB for 12 h. Data are mean ± SE (*n* = 4). Bars with a same letter are not significantly different at *P* < 0.05.

To examine levels of H_2_O_2_ in rice containing a hygromycin-detoxifying gene, we generated transgenic rice harboring *HPT*. *HPT* was overexpressed in three transgenic lines (OE-HPT-1, OE-HPT-2, OE-HPT-3), with no transcripts detected in non-transgenic wild-type (WT) plants (Figure 2A,B). Cultivation of seeds on a hydroponic solution containing 50 mg L^−1^ HyB showed that the three transgenic lines germinated and grew normally, but germination was inhibited in seeds from WT plants (Figure 2C). Numbers of germinated plants of WT and three transgenic lines were almost 100% without HyB treatment. In contrast, the germination rate of WT and the three transgenic lines was 0%, 72%, 68%, and 76%, respectively, when seeds were germinated on a hydroponic solution containing 50 mg L^−1^ HyB (Figure 2D). Upon treatment with HyB, H_2_O_2_ level significantly increased only in non-transgenic rice seedlings and not in the three transgenic lines containing *HPT* (Figure 3A). Then, we used DAB treatment to distinguish transgenic and non-transgenic rice. Without HyB treatment, leaf segments of both non-transgenic and transgenic rice showed very little brown color. Conversely, after DAB treatment, leaves of WT but not transgenic rice showed a dark brown color (Figure 3B). Moreover, HyB treatment significantly increased the production of malondialdehyde in WT but not OE-HPT-1 plants (Figure 3C), so HyB caused severe oxidative stress, thereby enhancing lipid peroxidation. 

### 2.3. DAB–H_2_O_2_ Method is Efficient for Detecting HPT-Containing Transgenic Rice

**Figure 4:**
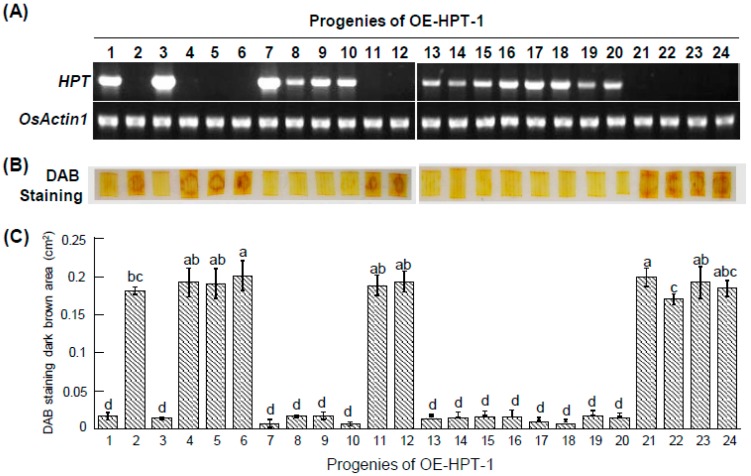
Detection of non-transformants and *HPT*-overexpressing transgenic rice by RT-PCR and DAB staining. (**A**) Total RNA was isolated from leaves of 24 progenies of the T1 segregation population of OE-HPT-1. Rice *Actin1* was an internal control. (**B**) H_2_O_2_ levels were detected by DAB staining in leaf segments of 24 T1 progeny of OE-HPT-1 treated with or without HyB for 12 h. (**C**) quantification of DAB staining results. The histogram tool in ImageJ was used to record the grayscale values of all pixels within the brown areas of eight-bit images. Each experiment was carried out in 4 technical replicates and 3 biological replicates. One representative experiment is shown. Data are mean ± SE. Bars with a same letter are not significantly different at *P* < 0.05.

To test the accuracy of the DAB–H_2_O_2_ method for distinguishing transgenic plants, we used RT-PCR and the DAB method to examine 24 T1 segregants from one transgenic plant carrying the *HPT* transgene (OE-HPT-1). On RT-PCR, *HPT* was detected in transgenic progenies but not null segregants (Figure 4A). T1 segregants without *HPT* transcripts showed significant DAB–H_2_O_2_ accumulation. Conversely, plants expressing *HPT* showed no significant DAB–H_2_O_2_ accumulation (Figure 4B). Thus, the DAB–H_2_O_2_ method was as reliable as RT-PCR for detecting transgenic plants containing an HyB-resistant gene.

### 2.4. Detection of Transgene-Free Genome-Edited Plants by DAB Method

To screen transgene-free genome-edited rice, the T1 population of *Agrobacterium*-mediated CRISPR/Cas9 rice was evaluated with the DAB method. The CRISPR/Cas9 binary vector encoded an *HPT* gene for transformation selection in the T0 generation [34,35]. After the CRISPR/Cas9 vector is used for gene editing at the target regions, the vector can be removed in the T1 segregation population. Four T1 genome-edited mutants (*osrr6/osrr11*#2-3-1, *osrr6/osrr11*#5-1-11, *osrr9/osrr10*#9-2-4, and *osrr9/osrr10*#10-3-6) from two transgenic events were used to examine the elimination of the transgene vector. The two transgenic events targeted multiple cytokinin two-component signaling type-A response regulators, OsRR6, OsRR11, OsRR9, and OsRR10. The mutations at the target region were verified by Sanger sequencing (Supplementary Table S1 [34,36]). The presence of the *HPT* selectable marker was visually selected by the DAB method and RT-PCR. 

**Figure 5:**
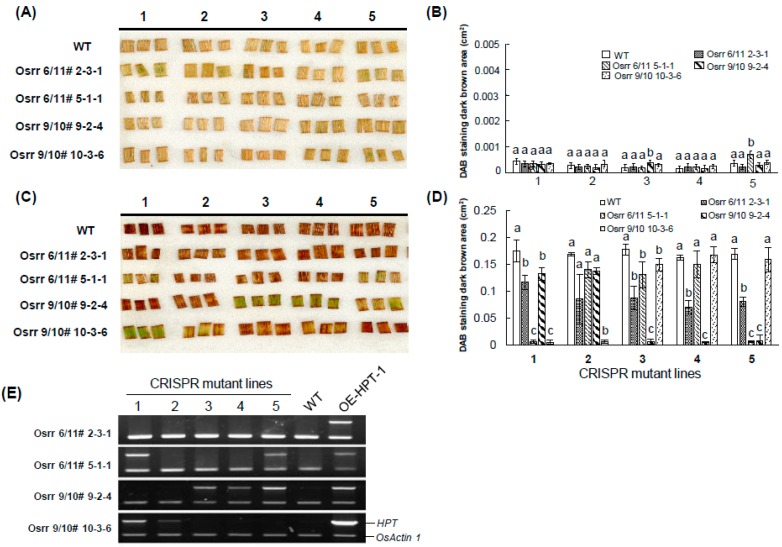
Detection of transgene-free CRISPR/Cas9 genome-edited plants by DAB staining in rice. Leaf painting assay and quantification of DAB staining results were carried out in leaf segments treated without (**A**,**B**) or with (**C**,**D**) 50 mg L^−1^ HyB, respectively. (**E**) Total RNA was isolated from leaves of CRISPR mutant lines (*osrr6/11*#2-3-1, *osrr6/11*#5-1-1, *osrr9/10*#9-2-4, and *osrr9/10*#10-3-6). *HPT* was detected by RT-PCR. *Rice Actin 1* was an internal control. Leaf segments of CRISPR mutant lines treated with or without HyB for 12 h were used for DAB staining. Each experiment was carried out in 4 technical replicates and 3 biological replicates. One representative experiment is shown. Data are mean ± SE. Bars with a same letter within each line are not significantly different at *P* < 0.05.

In *osrr6/osrr11* genome-edited lines, single guide RNAs (sgRNAs) specific to editing conserved sequences of OsRR6 and OsRR11 were designed. In *osrr9/osrr10* genome-edited lines, sgRNAs specific to editing conserved sequences of OsRR9 and OsRR10 were designed. No significant DAB–H_2_O_2_ accumulation was detected in leaves without HyB treatment (Figure 5A). After HyB treatment, a number of segregants of *osrr6/osrr11*#5-1-1, *osrr9/osrr10*#9-2-4, and *osrr9/osrr10*#10-3-6 showed slight DAB–H_2_O_2_ accumulation, which indicated the presence of *HPT*. In contrast, significant DAB–H_2_O_2_ accumulation could be detected in all tested segregants of *osrr6/11*#2-3-1, which strongly suggests that *osrr6/osrr11*#2-3-1 is a transgene-free homozygous null mutant (Figure 5A). 

Further RT-PCR analysis was conducted to validate the presence of the *HPT* gene in genome-edited lines. *HPT* could be detected in several segregants of *osrr6/11*#5-1-1, *osrr9/10*#9-2-4, and *osrr9/10*#10-3-6 (No. 1–5). In contrast, no *HPT* product could be detected in five segregants of *osrr6/11*#2-3-1, which indicates lack of the transgene in *osrr6/11*#2-3-1 (Figure 5C). However, segregants of genome-edited plants containing the *HPT* gene in *osrr6/11*#5-1-1 (No. 1 and 5), *osrr9/10*#9-2-4 (No. 3–5), and *osrr9/10*#10-3-6 (No. 1 and 2) all showed slight DAB–H_2_O_2_ staining (Figure 5B). In contrast, strong DAB–H_2_O_2_ accumulation was detected in segregants of genome-edited plants without the *HPT* gene: *Osrr6/11*#2-3-1 (No. 1–5), *osrr6/11*#5-1-1 (No. 2–4), *osrr9/10*#9-2-4 (No. 1 and 2), and *osrr9/10*#10-3-6 (No. 3–5). Hence, DAB–H_2_O_2_ staining is a reliable method for detecting transgenic plants free of a transgene (Figure 5B,C). No *HPT* product could be detected in WT plants. Transgenic OE-HPT-1 containing an *HPT* gene were a positive control.

### 2.5. HyB-Induced Overproduction of H_2_O_2_ Observed in Monocot and Dicot Plants

**Figure 6:**
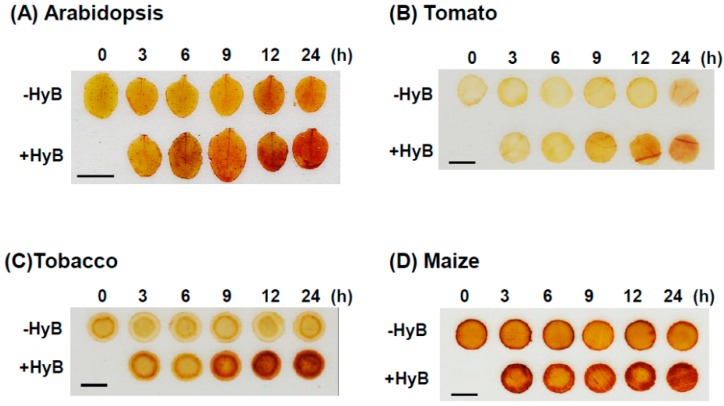
Detection of HyB-induced H_2_O_2_ accumulation by DAB staining in different plant species. (**A**) Arabidopsis leaves or leaf discs of (**B**) tomato, (**C**) tobacco, and (**D**) maize were treated with or without 50 mg L^−1^ HyB for different times as indicated. Ten leaf or leaf discs were used for DAB staining per sample, with 4 biological replicates for each treatment. Representative results are shown. Scale bars: 0.5 cm.

To examine whether H_2_O_2_ can be significantly induced by HyB in other plants, we examined plants such as *Arabidopsis*, tobacco, tomato, and maize. DAB–H_2_O_2_ staining showed that H_2_O_2_ was highly induced in all tested plants after HyB treatment (Figure 6). Tobacco showed the strongest accumulation of H_2_O_2_, followed by *Arabidopsis* and tomato. The monocot plant maize showed a slight enhancement of H_2_O_2_ after HyB treatment. 

## 3. Discussion

Genome-editing is a powerful tool for precision breeding in crops. Several countries will not regulate the use of genome-editing techniques in plants, which indicates that there will be more GE crops in the future. Therefore, developing a high-throughput screening platform for rapidly distinguishing transgenic or non-transgenic plants is required to efficiently generate transgene-free genome-edited plants. Our previous study showed that rice seedlings treated with HyB for 12 h markedly increased H_2_O_2_ level in non-transformants but not in transgenic rice containing the *HPT* gene [33]. In this study, we further verified that genome-edited rice progenies containing *HPT* can also be faithfully reflected by visual detection of the DAB–H_2_O_2_ complex in leaves and validated by RT-PCR, which indicates that the H_2_O_2_-based leaf painting assay is a rapid and reliable method. As compared with previous studies of cotton [29], rice [30], and maize [31], our H_2_O_2_-based method in rice largely decreased the time from five to seven days to 12 h, so the H_2_O_2_-based leaf painting assay is a high-throughput system for screening transgene-free genome-edited crops from segregating progenies.

## 4. Materials and Methods

### 4.1. Plant Materials

Rice (*Oryza sativa*, L. cv. Tainung 67) seedlings were grown hydroponically in half-strength Kimura B solution in a phytotron (Agricultural Experimental Station, National Taiwan University, Taipei, Taiwan) with natural light at 30 °C day/25 °C night and 90% relative humidity. *Arabidopsis thaliana* ecotype Columbia (Col-0), maize (*Zea mays* L. cv. Tainan-White), tobacco (*Nicotiana benthamiana* L.), and tomato (*Solanum lycopersicum* L. cv. Moneymaker) were grown in a mixture of perlite:vermiculite (1:1) in a growth chamber at 25 °C and light intensity 400 μmol m^−2^s^−1^ with a 16-h light/8-h dark cycle and relative humidity 80%. Two-week-old seedlings were used for HyB treatment. 

### 4.2. Generation of Hygromycinb (HyB)-Resistant Transgenic Rice

The *pPZP/HPH* binary vector [21] was used for transformation of HyB-resistant rice plants. The plasmid *pPZP/HPH* was introduced into *Agrobacterium tumefaciens* strain EHA105, and embryogenic calli derived from immature seeds of Tainung 67 were transfected as described [21]. Putative transformed calli were selected on HyB (Invitrogen, Carlsbad, CA, USA). Regenerated transgenic plants were grown and self-pollinated for two generations. For generating CRISPR/Cas9-mediated mutants, 20-bp sgRNAs complementary to rice cytokinin signaling type-A response regulators, *osrr6*, *osrr9*, *osrr10*, and *osrr11*, were designed and cloned into a pAS3 binary vector harboring an sgRNA cassette, a maize UBQ10-drived Cas9, and a hygromycin selection marker for *Agrobacterium*-mediated transformation [34,35,36]. The transformed calli were first selected on HyB media and the surviving calli were regenerated to T0 plantlets. At the T1 generation, the GE target regions may segregate with CRISRP/Cas9 vectors. To identify the mutants at the T1 generation, the sgRNA target sequences were amplified by PCR and subjected to one-step PAGE [37], high-resolution melt analysis (HRM) [38], or Sanger sequencing [39].

### 4.3. HyB Treatment

Leaf segments of 1 cm from 2-week-old rice seedlings were cut and cultured in sterile distilled water containing HyB. Leaf segments without any treatment were controls. For *Arabidopsis*, leaves of 2-week-old seedlings were treated with or without HyB. For tomato, tobacco, and maize, leaf discs were used for HyB treatment. All leaf segments/discs were treated with 50 mg L^−1^ HyB, and plants were incubated in a growth chamber at 27 °C under light for 12 h. We used 10 leaf segments or leaf discs per sample, with 4 biological replicates for each treatment. Representative results are shown.

### 4.4. Visual Detection of H_2_O_2_

H_2_O_2_ was visually detected in leaves by using DAB as a substrate [40]. Leaf segments or leaf discs were treated with or without 50 mg L^−1^ HyB. After 12 h, leaves were first rinsed with distilled water and then supplied with DAB solution (1 mg mL^−1^) through the cut ends for 12 h under light at 27 °C. Leaves were decolorized in boiling ethanol (95%) for 0.5 h. This treatment decolorized the leaves except for the brown polymerization. The H_2_O_2_ staining was repeated 4 times with similar results. 

### 4.5. Quantification of H_2_O_2_, DAB–H_2_O_2_, Malondialdehyde, and Protein Content

To measure H_2_O_2_ content, the reaction mixture consisted of 2 mL of 50 mM phosphate-buffered (pH 6.8) leaf extract supernatant and 1 mL reagent [0.1% (*v*/*v*) TiCl_4_ in 20% (*v*/*v*) H_2_SO_4_]. The blank reaction consisted of 50 mM phosphate buffer without leaf extract. H_2_O_2_ content was measured spectrophotometrically after a reaction with TiCl_4_ [41] with absorbance measured at 410 nm. The amount of H_2_O_2_ was calculated by using a standard curve prepared with known concentrations of H_2_O_2_ in some experiments, H_2_O_2_ content was measured spectrophotometrically after a reaction with DAB solution [42]. The reaction mixture consisted of 2 mL of 50 mM phosphate buffer (pH 6.8) leaf extract supernatant and 1 mg mL^−1^ DAB solution. The blank reaction consisted of 50 mM phosphate buffer without leaf extract. The absorbance was measured at 465 nm. To quantify DAB staining results, brown areas of the DAB–H_2_O_2_ reaction were scanned and measured with ImageJ (https://imagej.nih.gov/ij/). The ImageJ was used to record the grayscale values of all pixels within the brown areas of eight-bit images. An identical noise threshold was used for all analyses and was visually inspected for accurate representation of the particles. 

Malondialdehyde, routinely used as an indicator of lipid peroxidation, was extracted with 5% (*w*/*v*) trichloroacetic acid, and the content was determined by the thiobarbituric acid reaction as described [43] and expressed on the basis of fresh weight. For protein determination, leaves were homogenized in 50 mM sodium phosphate buffer (pH 6.8). Extracts were centrifuged at 17,600× *g* for 20 min, and the supernatant was used for determining protein level by the Bradford method [44].

### 4.6. RT-PCR Analysis

For molecular analysis of *HPT*-overexpressing transgenic rice and CRISPR mutant lines (*osrr6/osrr11*#2-3-1, *osrr6/orss11#*5-1-1, *osrr9/osrr10#*9-2-4, and *osrr9/osrr10#*10-3-6), total RNA was isolated by using TRIzol solution (Invitrogen, Carlsbad, CA, USA) from leaves of wild-type and putative transformants. For RT-PCR analysis, *HPT* was amplified with the primer sequences HPT-F, 5′-GTGCTTGACATTGGGGAGTT-3′, and HPT-R, 5′-ACATTGTTGGAGCCGAAATC-3′. PCR conditions were 94 °C for 5 min, then 32 cycles at 94 °C for 30 s, 50 °C for 30 s, and 72 °C for 1 min. Rice *Actin1* gene, amplified with the primer sequences Actin1-F, 5′-ATGCTCTCCCCCATGCTATC, and Actin1-R, 5′-TCTTCCTTGCTCATCCTGTC-3′, was an internal control. RT-PCR was performed in triplicate for each individual line; results from one repeat are shown in the figure. 

### 4.7. Statistical Analysis

Data are expressed as mean ± SE. Each experiment was carried out in 4 technical replicates and 3 biological replicates. One representative experiment is shown. Differences between measurements were analyzed by Student *t* test or Duncan’s multiple range test. *p* < 0.05 was considered statistically significant.

## 5. Conclusions

We show that the notable difference in H_2_O_2_ levels induced by HyB between non-transformant and transgenic rice harboring *HPT* can be a convenient system for distinguishing transgenic plants. Extraction of DNA or RNA is not necessary in this protocol. Instead, we used a simple DAB histochemical staining method, which was convenient, cheap, quick, and reliable for analysis of transgene-free genome-edited plants. The procedure was feasible for determining transgenic rice containing *HPT*. More importantly, HyB-induced overproduction of H_2_O_2_ in other plants demonstrated its broad application for a wide range of plant species.

## Supplementary Materials

The following are available online at https://www.mdpi.com/1422-0067/20/16/3885/s1.

## Abbreviations

**CRISPR**	Clustered Regularly Interspaced Palindromic Repeat
**DAB**	3,3′-diaminobenzidine
**HPT**	Hygromycin phosphotransferase
**HyB**	Hygromycin B
**WT**	Wild-type
