# Supplementary material for: H2O2-Based Method for Rapid Detection of Transgene-Free Rice Plants from Segregating CRISPR/Cas9 Genome-Edited Progenies
Source: Int J Mol Sci. 2019 Aug 9;20(16):3885. doi: 10.3390/ijms20163885 (PMC6720670; doi:10.3390/ijms20163885)
Supplement: Supplementary file 1 [file ijms-20-03885-s001.pdf]

Supplemental Table 1. Sequencing results of CRISPR-Cas9 generated rice mutants. The wild-type sequence is shown at the top with the PAM sequence highlighted in red boxes. Red dashes and red bold letters are deleted bases and inserted bases, respectively.

Gene	Genotype	Sequence Alignment
OsRR6	Wild type<br>OsRR6/11#2-3-1<br>OsRR6/11#5-1-11	401 CGGAGAACGTGCCGAACCGGATCAGCCGGTGCCTGGAGGAGGGCGCCGAGGAC 453<br>401 CGGAGAACGTGCCGAAC**A**CGGATCAGCCGGTGCCTGGAGGAGGGCGCCGAGGAC 454<br>401 CGGAGAACGTGCCGAA-CGGATCAGCCGGTGCCTGGAGGAGGGCGCCGAGGAC 452
OsRR11	Wild type<br>OsRR6/11#2-3-1<br>OsRR6/11#5-1-11	171 GGATAGCGGGAAGAGGGCCCTGGAACTGCTAGGCTCGGAACCAAATGTGAGCA 223<br>171 GGATAGCGGGAAGAGG-----------GCTAGGCTCGGAACCAAATGTGAGCA 212<br>171 GGATAGCGGGAAGAGGGCCCTGGAAC**T**TGCTAGGCTCGGAACCAAATGTGAGCA 224
OsRR9	Wild type<br>OsRR9/10#9-2-4<br>OsRR9/10#10-3-6	5 CAGTGGCTATAGAGGCTCCGTTCCATGTCCTGGCTGTGGATGATAGCCTT 54<br>5 CAGTGGCTATAGAGGCTCCGTTCCAT-TCCTGGCTGTGGATGATAGCCTT 53<br>5 CAGTGGCTATAGA-----------------TGGCTGTGGATGATAGCCTT 37
OsRR10	Wild type<br>OsRR9/10#9-2-4<br>OsRR9/10#10-3-6	5 CAGTGGCTATAGAGGCTCCGTTCCATGTCCTGGCTGTGGATGATAGCCTT 54<br>5 CAGTGGCTATAGAGGCTCCGTTCCAT-TCCTGGCTGTGGATGATAGCCTT 53<br>5 CAGTGGCTATAGAGGCTCCGTTCCAT-TCCTGGCTGTGGATGATAGCCTT 53
